# Diagnostic Agreement and 1‐Year Outcomes in Functional Neurological Disorder Following Neuroscience‐Informed Assessment, Education, and Counseling: A Retrospective Cohort Study

**DOI:** 10.1002/brb3.71491

**Published:** 2026-07-08

**Authors:** Adriano Mollica, Enoch Ng, Sricherry Nannapaneni, Unaisa Bhayat, Anthony Feinstein, David L. Perez, Matthew J. Burke

**Affiliations:** ^1^ Neuropsychiatry Program, Department of Psychiatry, Sunnybrook Health Sciences Centre University of Toronto Toronto Ontario Canada; ^2^ Hurvitz Brain Sciences Program Sunnybrook Research Institute Toronto Ontario Canada; ^3^ Temerty Faculty of Medicine University of Toronto Toronto Ontario Canada; ^4^ Department of Pediatrics Texas Tech University Health Sciences Center El Paso El Paso Texas USA; ^5^ Mass General Brigham Departments of Neurology and Psychiatry, Massachusetts General Hospital, Brigham and Women's Hospital Harvard Medical School Boston Massachusetts USA; ^6^ Division of Neurology, Department of Medicine University of Toronto Toronto Ontario Canada

**Keywords:** clinical outcomes, diagnostic agreement, functional neurological disorder, neuroscience‐informed education

## Abstract

**Background:**

We sought to explore outcomes at 1 year in functional neurological disorder (FND) following a neuroscience‐informed education and counseling assessment.

**Methods:**

Patients with FND were assessed at a quaternary neuropsychiatry clinic in Toronto, Canada, and provided education and counseling to build insight into their FND diagnosis. Patient‐determined diagnostic agreement at follow‐up was categorized as a binary variable: (i) symptoms attributable primarily to FND or (ii) attributable to another cause (neurological disease or unknown). One‐year symptom status was patient‐reported on a 7‐point scale (−3 to +3), with scores ≥2 defined as meaningful improvement. Return to work/school was assessed as an indicator of global improvement of functional status. Univariate tests screened variables for inclusion in multivariate logistic regression models, which evaluated associations between diagnostic agreement and other outcomes.

**Results:**

A total of 282 patients with FND were assessed (mean age 38.9 ± 12.0 years; 80.3% female), and of these, 127 had 1‐year follow‐up data. FND subtypes included functional movement disorder (functional weakness [26.8%], hyperkinetic movement [15.7%]), seizure (15.0%), sensory (16.5%), functional cognitive disorder (7.9%), persistent postural perceptual dizziness (14.2%), and speech/swallowing (3.9%). Diagnostic agreement was significantly associated with symptom improvement (odds ratio [OR] = 3.81, 95% CI: 1.33–11.71, *p *= 0.015) and global improvement (OR = 5.74, 95% CI: 1.11–37.54, *p *= 0.047). Psychiatric comorbidity (*p *= 0.026), childhood trauma (*p *= 0.015), and psychological triggers (*p *= 0.013) were associated with diagnostic agreement, while ongoing medical/neurological workup was linked to disagreement (*p* < 0.001).

**Conclusion:**

Diagnostic agreement was associated with symptom improvement and return‐to‐work/school status in FND patients who received a neuroscience‐informed education and counseling assessment. However, given the observational design and lack of baseline measurement of diagnostic agreement, the directionality of this relationship cannot be determined.

## Graphical abstract

This retrospective cohort study examined one‐year outcomes in 282 patients with Functional Neurological Disorder (FND) following a neuroscience‐informed neuropsychiatry assessment with education and counseling. At one‐year follow‐up (N = 127), 40% showed moderate‐to‐strong symptom improvement and 44% showed global functional improvement. Two‐thirds of patients (66%) agreed with their FND diagnosis, and diagnostic agreement was independently associated with improved symptom status and return to work or school, suggesting that neuroscience‐informed education may support diagnostic acceptance and better outcomes in FND.

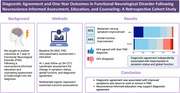

## 1 Introduction

Functional neurological disorder (FND) is a complex neuropsychiatric condition encompassing a broad range of symptom presentations that are incompatible with other recognized medical/neurological conditions. Substantial progress has been made in establishing “positive” clinical signs (e.g., Hoover's sign, tremor entrainment) of FND that are the basis for diagnosis (Espay et al. 2018; Aybek and Perez 2022). FND has an estimated prevalence of 80 per 100,000 and is likely among the top five reasons for outpatient neurological consultation (Espay et al. 2018; Finkelstein et al. 2025; Stone et al. 2010). This condition has high rates of disability, substantial healthcare costs, and limited available evidence‐based treatment options (Espay et al. 2018; Barsky et al. 2005; Gelauff et al. 2014; Stephen et al. 2025). Communicating the diagnosis in a transparent and empathic manner is a critical first step in management (Stone et al. 2020; Stone et al. 2016).

Perhaps intuitively, diagnostic agreement is likely an important step to facilitate participation in FND care pathways. Prior studies have shown that belief in the diagnosis is associated with greater treatment engagement, reduced symptom severity, and improved outcomes (LaFaver et al. 2021; Cope et al. 2021; Greenfield et al. 2024; Asadi‐Pooya et al. 2020). In contrast, diagnostic disagreement has been linked to resistance to treatment and poorer outcomes, particularly when the diagnosis is poorly communicated or not accepted (Gilmour et al. 2024; Salmon et al. 1999). Yet, diagnostic agreement remains understudied in FND, particularly as it pertains to outcomes. Beyond diagnostic agreement, other neuropsychiatric factors linked to outpatient prognosis of FND are incompletely understood, although there is emerging evidence that psychiatric and medical comorbidities, illness duration, and select psychosocial factors (e.g. disability payment or litigation) may have prognostic implications for the motor and seizure subtypes of FND (Gelauff et al. 2014; LaFrance and Syc 2009; Gelauff et al. 2019; Duncan et al. 2016; Glass et al. 2018). However, reported outcomes have been limited by exclusion criteria (e.g., clinics that do not accept patients with common comorbidities) and/or focus on specific FND subpopulations (e.g., only patients with motor FND) (Gelauff et al. 2014; Gelauff et al. 2019), whereas there is emerging recognition of other nonmotor subtypes of FND (e.g., speech–language problems, chronic dizziness, vision, and cognition) (Espay et al. 2018; Hallett et al. 2022).

The FND Neuropsychiatry Clinic at our academic center provides a unique opportunity to collect and evaluate data addressing these gaps due to the broad inclusion criteria of all FND subtypes/presentations and comorbidities. Here, we retrospectively examine a cohort of FND patients who received a comprehensive neuroscience‐informed education and counseling session at their initial assessment and explore relationships between baseline variables and 1‐year outcomes, such as diagnostic agreement, symptom status, and global function. Our main hypothesis was that diagnostic agreement (i.e., patients accepting the FND diagnosis) would be associated with better symptom and global outcomes.

## 2 Methods

### 2.1 Study Design and Setting

This retrospective cohort study was conducted at the FND Clinic at Sunnybrook Health Sciences Centre (SHSC), a quaternary neuropsychiatry clinic led by a neurologist in Toronto, Ontario, Canada. A total of *n* = 282 consecutive patients were seen in the clinic between July 1, 2019, and June 1, 2024. Of these, 127 patients had 1‐year follow‐up data. Patients with any FND subtype (e.g., motor, seizure, cognitive, sensory, dizziness, or speech/swallowing presentations) were eligible for inclusion (Hallett et al. 2022). Due to the COVID‐19 pandemic and the clinic's large geographic catchment area, the majority of patient visits were conducted via video telehealth.

### 2.2 Ethics and Confidentiality

This study received ethics approval from the Sunnybrook Research Ethics Board. A waiver of consent was obtained as the study involved minimal risk and relied on retrospective data analysis.

### 2.3 Patient Population and Interventions

Inclusion criteria included adult patients (>18 years old) seen for consultation in the FND clinic and who had completed a follow‐up appointment at 1 year. Generally, all patients whose core symptoms were due to FND were offered follow‐up. Patients without FND as a primary explanation of their symptoms (e.g., somatic symptom disorder, psychotic disorder with somatic delusions, chronic pain syndrome) were excluded.

All patients received neuroscience‐based education and counseling to build insight into their FND diagnosis (see Supporting Information for an example). Patients also received a personalized biopsychosocial formulation bringing together their predisposing, precipitating, and perpetuating factors pertaining to their FND symptoms. Emphasis was placed on presenting FND as common, real, and treatable, as well as leveraging distraction/attention‐redirection strategies and encouraging gradual re‐engagement with potential avoided activities/hobbies/work/school. Counseling and education were provided in tandem with recommendations for management of their FND symptoms and multidisciplinary services in the community as clinically indicated. This was all done as part of a single initial consultation assessment. Unfortunately, there was no in‐house treatment available for patients.

### 2.4 Data Collection

Baseline data were collected retrospectively through manual extraction from electronic medical records (A.M. and U.B.), drawing on both structured fields and clinician free‐text documentation. While all patients were assessed by the same clinician at follow‐up, initial consultations were completed by a clinical team, with different learners (e.g., fellows, residents) contributing to history‐taking and note documentation. Data extraction was guided by a standardized consensus table approved by the lead authors (A.M. and M.B.), which prespecified all variables of interest. Any discrepancies or uncertainties were reviewed and resolved by the study team through mutual agreement. As expected in retrospective chart review, documentation variability resulted in missing data for some variables. Variables with substantial missing information or limited reliability were excluded from descriptive and inferential analyses.

One‐year follow‐up data were collected systematically using a standardized clinical template administered at each follow‐up visit by the same clinician (M.B.). This template included core outcomes (e.g., diagnostic agreement, symptom change, functional status) as well as additional follow‐up variables such as perceived symptom trigger/etiology and engagement in rehabilitation‐based interventions (e.g., psychotherapy, physiotherapy). Participation in psychotherapy and/or physiotherapy was recorded based on patient report and documentation at follow‐up; while no formal minimum “dose” was prespecified a priori, participation generally reflected engagement in at least several sessions rather than a single encounter.

### 2.5 Outcome Measures

Main outcomes of interest included diagnostic agreement, symptom status, and global function status (as indicated by return to work/school). Outcomes were assessed by the same clinician at the 1‐year follow‐up since the initial consultation.

Diagnostic agreement was patient‐determined only at the 1‐year follow‐up visit and categorized as a binary variable: (i) symptoms attributable primarily to FND or (ii) attributable to another cause (e.g., other structural or neurological disease or unknown). Determination of agreement was made in relation to the patient's presenting functional neurological symptoms, which had been diagnosed based on the presence of positive rule‐in clinical signs of FND. Patients were asked an open‐ended question about their illness beliefs (i.e., “What diagnosis do you believe is/was causing your [insert main presenting FND symptom(s)]?”), and their responses were categorized accordingly. Of note, while a subset of patients had neurological or medical comorbidities, the “other structural or neurological disease” category was if they attributed their primary functional neurological symptom(s) to another etiology (i.e., not FND).

Symptom status was evaluated using a 7‐point patient‐reported scale (−3 = *strongly worse*, 0 = *no change*, to +3 = *full resolution*), which has been previously used in retrospective cohort studies evaluating outcomes in FND (Glass et al. 2018). For regression analyses related to symptom outcome, scores of +2 or higher were classified as “clinically meaningful improvement,” reflecting a more stringent threshold for improvement. Scores of ≤1 were grouped as “not substantially improved or worse,” based on the rationale that +1 represented only minimal improvement. This binarized approach allowed for a clearer distinction between clinically meaningful improvement and lack of significant progress.

The secondary objective was to assess change in global function at 1 year. Improvement in global function was defined as a positive change from being off work at baseline due to FND symptoms to being engaged in part‐ or full‐time work or school at 1‐year follow‐up. Patients who maintained their work or school status between baseline and 1‐year follow‐up were not included in this analysis.

### 2.6 Statistical Analysis

Descriptive statistics summarized clinical characteristics at baseline and 1‐year follow‐up. Ordinal logistic regression examined the association between diagnostic agreement and ordinal symptom status levels (−3 to +3) at 1 year. Univariate screening tests (chi‐square, *t*‐tests for variables with normal distribution; Wilcoxon rank sum test for variables with nonnormal distribution; and logistic regression) were conducted to identify variables associated with symptom and work/school outcomes. Candidate variables for multivariate logistic regression were identified using univariate screening analyses, with variables meeting a prespecified threshold of *p* < 0.10 retained for multivariable modeling to evaluate variables independently associated with clinically meaningful symptom and work/school improvement (Hosmer et al. 2013). Multivariate logistic regressions were the definitive statistical test for our results. For logistic regression, a binary outcome was used for symptom status (scores ≥2 = significant improvement, <2 = worse or no/minimal improvement). This threshold was selected based on clinical relevance and sample size constraints, as multivariate ordinal logistic regression was not feasible with the available data. A threshold of *p* < 0.05 was used to determine significance in the multivariate models. Wilcoxon rank sum tests were used to assess differences in symptom status changes based on diagnostic agreement for those who participated in psychotherapy and physiotherapy. Model diagnostics included assessment of multicollinearity using the variance inflation factor and overall model fit using McFadden's pseudo‐*R* ^2^. All statistical analyses were conducted using R version 3.6.3. Given the retrospective observational design, analyses were intended to explore associations between diagnostic agreement and clinical outcomes; causal inferences cannot (and should not) be made.

## 3 Results

### 3.1 Baseline Characteristics

The full cohort included *n* = 282 patients with FND (mean age 40 ± 13 years; 78% female) (Table 1). The average symptom duration at baseline was 3.6 ± 5.5 years (median = 2.0), with a mean of 4.4 ± 2.4 concurrent functional symptoms. Of these, *n* = 127 (45%) had 12‐month follow‐up data available (mean age 39 ± 12 years; 80% female). There were no significant baseline differences between patients with and without follow‐up on demographic or clinical characteristics, except for migraine history, which was more common in those with follow‐up (31% vs. 18%, *p* = 0.008).

**TABLE 1:** Baseline demographic and clinical characteristics of patients with functional neurological disorder, comparing those with and without 12‐month follow‐up.

Characteristic	Overall (*N* = 282)	No follow‐up (*N* = 155)	1‐year follow‐up (*N* = 127)	Uncorrected *p*‐value^a^
Demographics				
Age (mean ± SD)	40 ± 13	40 ± 14	39 ± 12	0.626
Female sex	221 (78%)	119 (77%)	102 (80%)	0.567
Married/partnered	120 (48%)	65 (44%)	55 (55%)	0.136
Duration of illness (mean ± SD)	3.6 ± 5.5	3.5 ± 5.7	3.7 ± 5.3	0.142
Receiving disability benefits	82 (30%)	45 (30%)	37 (29%)	0.999
Ongoing litigation	17 (6%)	10 (6%)	7 (6%)	0.940
On leave from work/school at baseline	150 (54%)	82 (54%)	68 (54%)	0.904
FND subtype				
Hyperkinetic motor^b^	54 (19%)	33 (21%)	21 (17%)	0.154
Tremor	12 (4%)	8 (5%)	4 (3%)	
Dystonia	5 (2%)	3 (2%)	2 (1%)	
Tics	2 (1%)	2 (1%)	0 (0%)	
Gait	14 (5%)	11 (7%)	3 (2%)	
Jerks	21 (7%)	10 (7%)	11 (9%)	
Hypokinetic motor	70 (25%)	37 (24%)	33 (26%)	
Seizure	43 (15%)	24 (15%)	19 (15%)	
Sensory	50 (18%)	29 (18%)	21 (17%)	
Cognitive	27 (10%)	17 (11%)	10 (8%)	
PPPD	24 (9%)	6 (4%)	18 (14%)	
Speech/swallowing	14 (5%)	9 (6%)	5 (4%)	
Symptom domains at baseline				
Cognitive symptoms	146 (52%)	74 (48%)	72 (57%)	0.147
Fatigue	111 (40%)	56 (37%)	55 (44%)	0.277
Body pain	120 (43%)	68 (44%)	52 (41%)	0.751
Headache	108 (39%)	54 (36%)	54 (43%)	0.261
Triggers for symptom onset				
Physical stressor	140 (50%)	76 (49%)	64 (51%)	0.862
Psychological stressor	114 (41%)	58 (37%)	56 (46%)	0.214
Lifetime comorbidities and other health factors				
Neurological comorbidity^c^	145 (51%)	77 (50%)	68 (54%)	0.256
Head injury	69 (25%)	42 (27%)	27 (22%)	0.331
Post‐concussion syndrome	43 (15%)	27 (17%)	16 (13%)	0.404
Migraine headaches	68 (24%)	28 (18%)	40 (31%)	0.008
Fibromyalgia	18 (7%)	8 (5%)	10 (8%)	0.337
Chronic pain	53 (19%)	30 (19%)	23 (19%)	0.999
Irritable bowel syndrome	28 (10%)	12 (8%)	16 (13%)	0.164
Chronic fatigue	11 (4%)	7 (5%)	4 (3%)	0.760
Lifetime psychiatric history				
Any psychiatric history	192 (68%)	104 (67%)	88 (69%)	0.791
Anxiety disorder	135 (48%)	68 (44%)	67 (54%)	0.120
Major depressive disorder	119 (43%)	62 (40%)	57 (46%)	0.396
Bipolar disorder	13 (5%)	9 (6%)	4 (3%)	0.396
PTSD	51 (18%)	28 (18%)	23 (18%)	0.999
ADHD	25 (9%)	15 (10%)	10 (8%)	0.675
Developmental disorder	12 (4%)	8 (5%)	4 (3%)	0.556
Psychotic disorder	4 (1%)	3 (2%)	1 (1%)	0.630
Personality disorder	38 (13%)	21 (14%)	17 (13%)	0.968
Substance use disorder	46 (17%)	27 (18%)	19 (15%)	0.629
Active psychiatric symptoms at baseline				
Depressed mood	95 (34%)	57 (37%)	38 (30%)	0.264
Anxiety symptoms	169 (61%)	91 (58%)	78 (64%)	0.363
Obsessive–compulsive symptoms	3 (1%)	1 (1%)	2 (2%)	0.590
PTSD symptoms	24 (9%)	14 (9%)	10 (8%)	0.831
Psychotic spectrum symptoms	4 (1%)	3 (2%)	1 (1%)	0.630
Dissociative symptoms	19 (7%)	7 (5%)	12 (10%)	0.150
Suicidal ideation	29 (10%)	15 (10%)	14 (11%)	0.844
Trauma history				
Childhood trauma	146 (67%)	92 (67%)	54 (68%)	0.917
History of any physical abuse	64 (26%)	27 (20%)	37 (32%)	0.210
History of any sexual abuse	62 (25%)	29 (21%)	33 (29%)	0.404

^a^ Wilcoxon rank sum test used for age and duration of illness (nonnormal distribution); Pearson's chi‐squared test used for categorical variables, with Fisher's exact test applied when any expected cell count was <5.

^b^ Includes tremor, dystonia, tics, gait, and jerks.

^c^ Includes epilepsy, multiple sclerosis, stroke, peripheral neuropathy, spinal stenosis, and benign brain tumors.

Abbreviations: ADHD, attention‐deficit hyperactivity disorder; PTSD, posttraumatic stress disorder.

FND subtypes included motor hypokinetic (27%), motor hyperkinetic (16%), seizure (15%), sensory dominant (17%), functional cognitive (8%), PPPD (14%), and speech/swallowing (4%). Within motor hyperkinetic FND (*n* = 54), presentations included tremor (*n* = 12), dystonia (*n* = 5), tics (*n* = 2), gait disorder (*n* = 14), and jerks/myoclonus (*n* = 21) (Table 1).

Lifetime neurological comorbidities (e.g., multiple sclerosis, epilepsy, stroke) were present in 16%, and 32% had a migraine history. Lifetime psychiatric comorbidities were reported in 69%, most commonly anxiety disorders (53%) and major depressive disorder (45%), followed by PTSD (18%), personality disorder (13%), substance use disorder (10%), and attention‐deficit/hyperactivity disorder (8%). The mean number of lifetime psychiatric diagnoses per patient was 1.6 (range: 1–7). Current low mood and anxiety/panic were reported in 30% and 63% at baseline, respectively.

Chronic pain conditions were documented in 31%, including fibromyalgia (8%), irritable bowel syndrome (13%), and other chronic pain syndromes (19%). Persistent post‐concussion symptoms and chronic fatigue syndrome were noted in 13% and 3%, respectively.

### 3.2 One‐Year Follow‐Up Characteristics

Follow‐up rates at 12 months by baseline FND subtype are provided in Table 1.

At follow‐up, 66% reported agreement with their diagnosis (i.e., FND as the primary explanation for their presenting symptom) (Table 2). Comparatively, 34% had varying levels of disagreement with 15% believing another structural condition was responsible for their symptoms in addition to FND, 14% believed symptoms were entirely due to another structural condition and not FND, and 6% thought their symptoms were unexplained. Triggers for onset of illness varied and were reported as physical (25%), psychological (38%), both physical and psychological (25%), or unknown (13%).

**TABLE 2:** Summary of patient characteristics and outcomes at baseline and 1‐year follow‐up based on diagnostic agreement.

Variable	Total (*N* = 127)	Disagreement (*N* = 42, 34%)	Agreement (*N* = 85, 66%)	Uncorrected *p*‐value
Demographics				
Age (mean ± SD)	39 ± 12	41 ± 12	38 ± 12	0.25
Female sex	102 (80%)	35 (84%)	67 (79%)	0.72
Illness duration (mean ± SD)	3.7 ± 5.3	4.7 ± 7.3	3.3 ± 3.8	0.26
Number of symptoms (mean ± SD)	5.4 ± 2.4	5.7 ± 2.4	5.2 ± 2.4	0.31
On leave from work/school	69 (54%)	21 (49%)	48 (56%)	0.61
Lifetime comorbidities and other health factors				
Neurological comorbidity	20 (16%)	7 (16%)	14 (16%)	0.99
Chronic pain	24 (19%)	7 (16%)	17 (20%)	0.20
Migraine	41 (32%)	14 (33%)	27 (32%)	0.99
Fibromyalgia	10 (8%)	4 (9%)	7 (8%)	0.99
IBS	17 (13%)	6 (14%)	11 (13%)	0.99
PCS	17 (13%)	5 (12%)	11 (13%)	0.99
Chronic fatigue	4 (3%)	1 (2%)	3 (4%)	0.99
Other FSD	9 (7%)	3 (7%)	6 (7%)	0.99
Abnormal brain imaging	17 (13%)	6 (14%)	10 (12%)	0.99
Lifetime psychiatric history				
Any psychiatric comorbidity	88 (69%)	24 (56%)	65 (77%)	0.03
Anxiety disorder	67 (53%)	18 (44%)	50 (59%)	0.15
PTSD	23 (18%)	4 (9%)	20 (24%)	0.09
Mood disorder	61 (48%)	16 (37%)	45 (53%)	0.13
Active psychiatric symptoms at baseline				
Depressed mood	38 (30%)	14 (33%)	23 (27%)	0.67
Anxiety symptoms	80 (63%)	26 (63%)	55 (65%)	0.73
Trauma history				
Childhood trauma	86 (68%)	21 (51%)	65 (76%)	0.01
Emotional abuse	55 (43%)	13 (32%)	41 (48%)	0.17
Physical abuse	25 (20%)	5 (11%)	20 (24%)	0.16
Sexual abuse	39 (31%)	9 (22%)	30 (35%)	0.23
FND subtype^a^				0.08
Hyperkinetic motor	20 (16%)	6 (30%)	14 (70%)	
Hypokinetic motor	34 (27%)	11 (32%)	23 (68%)	
Seizure	19 (15%)	4 (21%)	15 (79%)	
Sensory	20 (16%)	9 (45%)	11 (55%)	
Cognitive	10 (8%)	3 (30%)	7 (70%)	
PPPD	17 (14%)	6 (35%)	11 (65%)	
Speech/swallowing	5 (4%)	0 (0%)	5 (100%)	
One‐year follow‐up data				
Ongoing structural workup	29 (23%)	24 (56%)	5 (6%)	<0.001
Receiving disability payment	37 (29%)	11 (26%)	27 (32%)	0.61
Ongoing litigation	11 (9%)	4 (9%)	8 (9%)	0.76
Participated in psychotherapy	89 (70%)	26 (63%)	63 (74%	0.25
Participated in physiotherapy	53 (42%)	18 (44%)	33 (39%)	0.69
Symptom status				0.003
*Strongly worse* (−3)	2 (2%)	1 (1%)	1 (1%)	
*Moderately worse* (−2)	2 (2%)	2 (5%)	0 (0%)	
*Mildly worse* (−1)	11 (9%)	8 (19%)	2 (2%)	
*No change* (0)	17 (13%)	7 (16%)	10 (12%)	
*Mildly better* (1)	23 (18%)	9 (21%)	14 (17%)	
*Moderately better* (2)	51 (40%)	15 (35%)	35 (43%)	
*Strongly better* (3)	21 (17%)	1 (2%)	20 (24%)	
Global status^b^				
Improvement	56 (44%)	13 (31%)	43 (50%)	0.02
Unchanged	69 (54%)	31 (69%)	38 (45%)	
Worsened	3 (2%)	2 (5%)	1 (1%)	
Perception of illness trigger				0.01
Physical	27 (25%)	17 (40%)	29 (34%)	
Psychological	46 (38%)	5 (12%)	31 (38%)	
Both (physical and psychological)	36 (25%)	14 (33%)	13 (16%)	
Unexplained	15 (13%)	6 (14%)	9 (11%)	

^a^ Hyperkinetic motor includes functional tremor, jerks, tics, or gait. Hypokinetic motor includes only functional weakness.

^b^ Defined as a transition from not working at baseline to working part‐ or full‐time at follow‐up.

Symptom status at 1‐year follow‐up was as follows: strong worsening (−3, 2%), moderate worsening (−2, 2%), mild worsening (−1, 9%), no change (0, 13%), mild improvement (+1, 18%), moderate improvement (+2, 40%), and strong improvement (+3, 17%). Among the 127 patients with 1‐year follow‐up data, 68 (54%) were off work or school at baseline due to their symptoms. Global improvement in work or school status, defined as a transition from not working at baseline to working part‐ or full‐time at follow‐up, was observed in 56 (44%) of patients, while 69 (54%) remained unchanged and three (2%) worsened.

In terms of treatment, 70% pursued various forms of psychotherapy (e.g., supportive therapy, cognitive behavioral therapy, psychodynamic psychotherapy, trauma therapy etc.) and 42% participated in physiotherapy.

### 3.3 Univariate Screening Results

**FIGURE 1:**
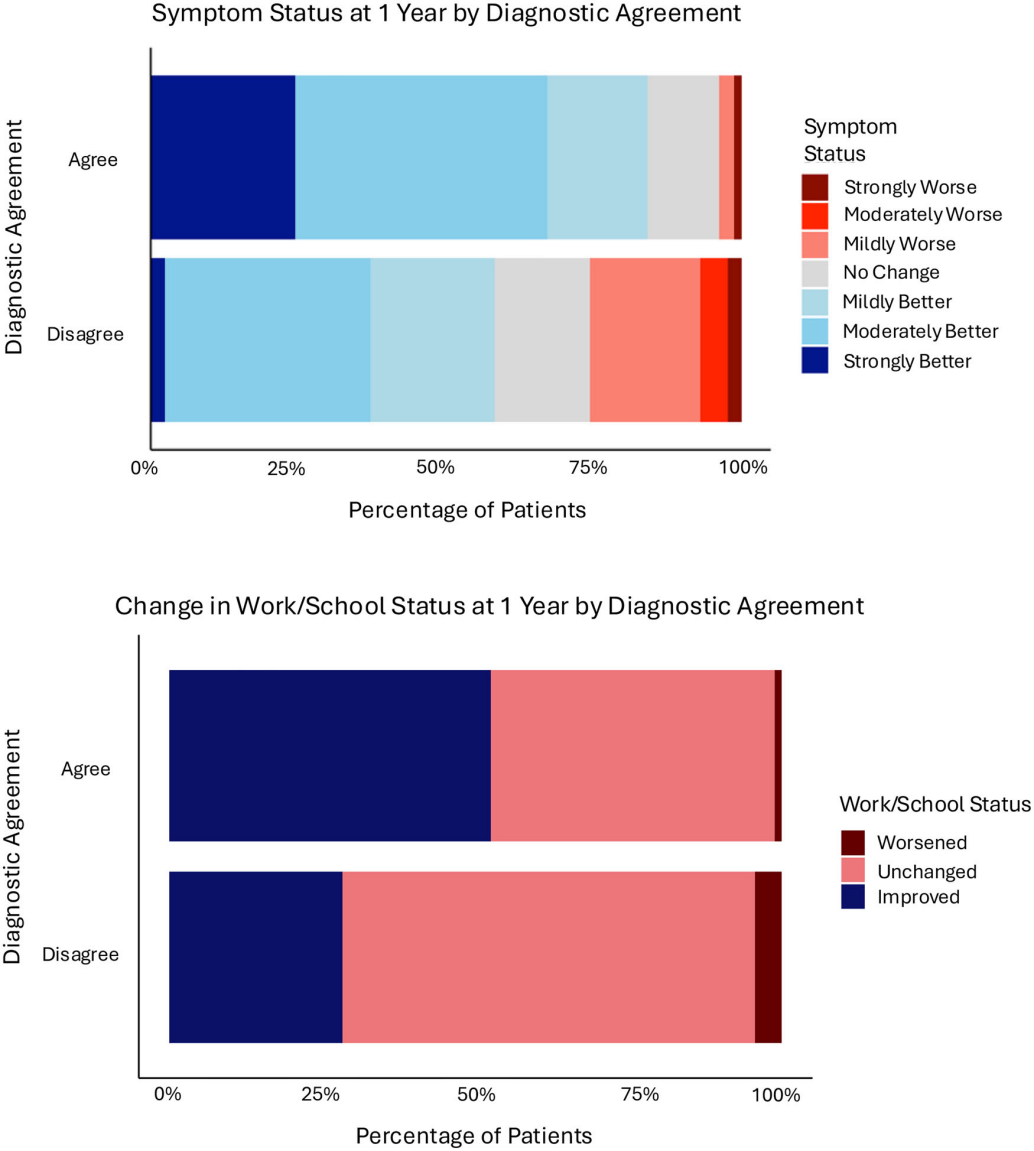
Relationship between diagnostic agreement and 1‐year outcomes. Top panel: Distribution of symptom status at 1 year by diagnostic agreement. Patients who agreed with their FND diagnosis at follow‐up were also more likely to report improvement, with diagnostic agreement associated with over fourfold increased odds of better outcomes in univariate ordinal logistic regression (OR = 4.44, *p* < 0.001). Bottom panel: Change in work or school status at 1 year by diagnostic agreement. Diagnostic agreement was significantly associated with improvement in univariate screening analyses (*p* = 0.023) and remained significant in multivariate modeling (*p* = 0.047).

To test the hypothesis that diagnostic agreement co‐varies with outcomes, an ordinal logistic regression was conducted using the full range of symptom status levels (–3 to +3). Patients who agreed with the diagnosis had significantly greater odds of symptom improvement at 1 year (odds ratio [OR] = 4.44, 95% CI: 2.19–8.99, *β* = 1.49, SE = 0.36, *p* < 0.001; Figure 1).

For binary outcomes (scores ≥2 = clinically meaningful improvement, <2 = worse or no/minimal improvement), diagnostic agreement (*p* = 0.003), perceived psychological trigger for illness onset (*p* = 0.032), and history of sexual trauma (*p* = 0.042) were significantly associated with symptom improvement. Baseline depressed mood (*p* = 0.012) and ongoing litigation (*p* = 0.049) were significantly associated with poorer outcomes.

Diagnostic agreement was also significantly associated with global status at 1 year, with patients who agreed with the diagnosis more likely to demonstrate improvement relative to those who disagreed (*p* = 0.023; Figure 1).

Younger age was associated with greater likelihood of improvement in work or school status at 1 year (*p* = 0.04). No other variables were independently associated with change in global status (Table 3).

**TABLE 3:** Nonsignificant univariate screening results for symptom status and global function status at 1‐year.

Variable	*p*‐value
Symptom status	
Age	0.63
FND subtype	0.46
Baseline symptom count	0.61
Perception of illness trigger	0.33
Ongoing structural workup	0.56
History of sexual trauma	0.06
Litigation status	0.09
Global status	
Baseline symptom count	0.65
Baseline depressed mood	0.48
History of sexual trauma	0.08
Perception of illness trigger	0.07
Ongoing structural workup	0.92
Litigation status	0.21
FND subtype	0.99

Psychotherapy and physiotherapy participation during the follow‐up period were not significantly associated with changes in either symptom or global status.

### 3.4 Multivariate Results

#### 3.4.1 Symptom Improvement at 1 Year

Diagnostic agreement (OR = 3.81, 95% CI: 1.33–11.71, *p* = 0.015) and absence of receiving disability payment (e.g., long‐term disability) (OR = 2.74, 95% CI: 1.12–7.26, *p* = 0.033) were significantly associated with clinically meaningful symptom improvement (scores ≥2). Baseline depressed mood was also independently associated with lower odds of improvement (OR = 0.34, 95% CI: 0.12–0.91, *p* = 0.035).

#### 3.4.2 Work/School Status Improvement at 1 Year

Agreement with the FND diagnosis (OR = 5.74, 95% CI: 1.11–37.54, *p* = 0.047) and younger age (OR = 0.91 per year increase, 95% CI: 0.83–0.99, *p* = 0.04) were independently associated with improvement in work or school status at 1 year.

#### 3.4.3 Model Diagnostics

Both multivariate logistic regression models demonstrated good overall fit (McFadden's *R* ^2^ = 0.21 for symptom status, McFadden's *R* ^2^ = 0.32 for work/school status). Variance inflation factors were all <2, indicating no concerns of multicollinearity.

### 3.5 Variables Associated With Diagnostic Agreement

A positive history of psychiatric comorbidity (*p* = 0.026), childhood trauma (*p* = 0.015), and perception of a psychological trigger for illness (*p* = 0.013) were significantly associated with diagnostic agreement. Patients who underwent additional structural workup during interval follow‐up were significantly less likely to agree with the FND diagnosis (*p* < 0.001). No significant associations were found with disability payment status (*p* = 0.61), litigation (*p* = 0.76), abnormal brain imaging (*p* = 0.99), neurological comorbidity excluding migraine or concussion (*p* = 0.99), biological sex (*p* = 0.72), or presenting symptom subtype (*p* = 0.11). Similarly, no significant differences were observed for age (*p* = 0.25), number of baseline symptoms (*p* = 0.31), or illness duration (*p* = 0.26).

**FIGURE 2:**
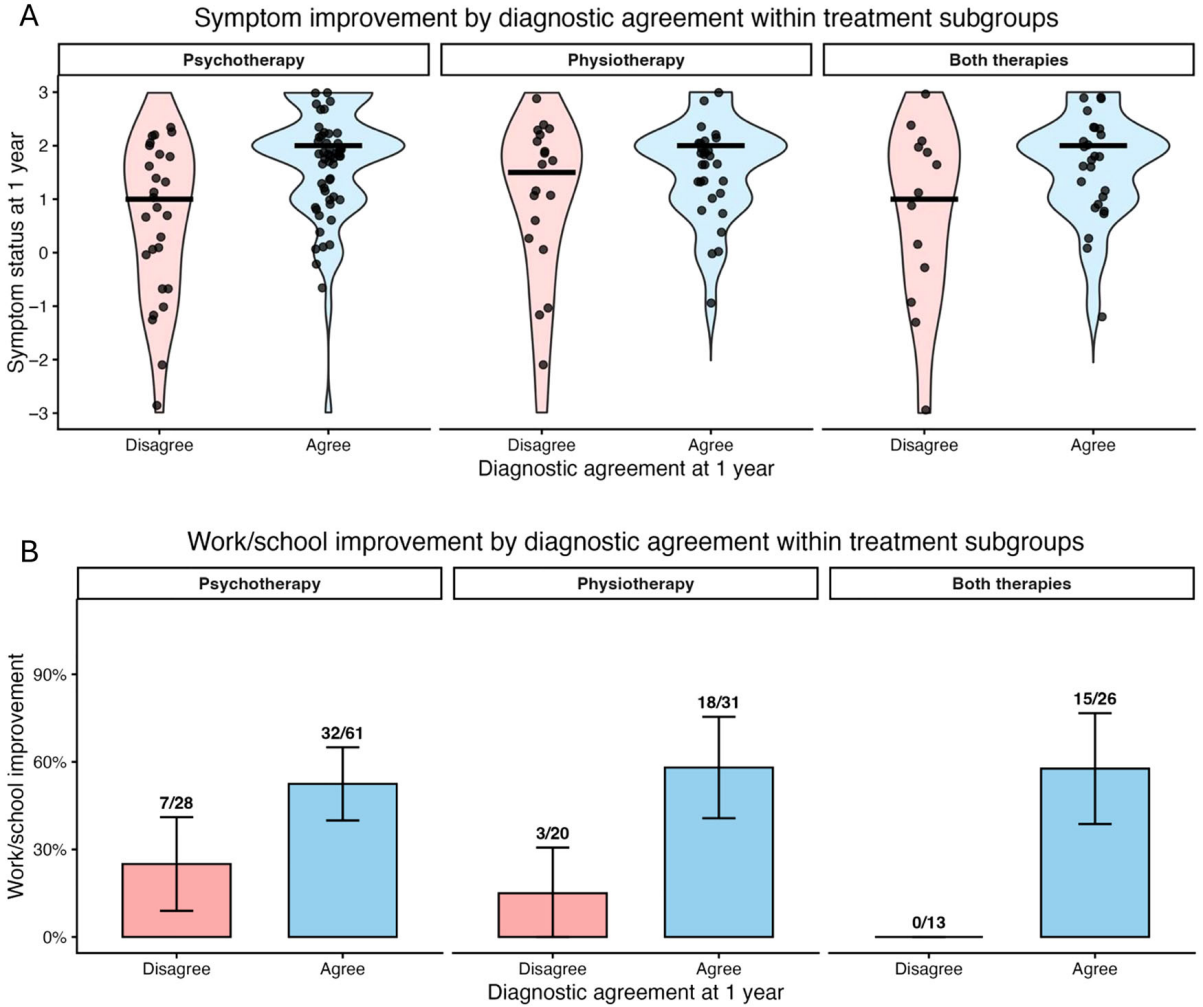
Symptom status at 1 year based on diagnostic agreement among patients engaging in psychotherapy and physiotherapy. Top panel (A) shows scatter–violin plots of symptom status at 1 year (ordinal scale −3 to +3) by diagnostic agreement among patients receiving psychotherapy (*n* = 89), physiotherapy (*n* = 51), or both therapies (*n* = 39). Diagnostic agreement was associated with greater symptom improvement in the psychotherapy subgroup (Wilcoxon *p* < 0.001, *r* = 0.37) but not in the physiotherapy (*p* = 0.17) or combined therapy (*p* = 0.15) subgroups. Bottom panel (B) shows the proportion of patients with improvement in work/school status (bars represent proportions with 95% confidence intervals). Diagnostic agreement was associated with work/school improvement across treatment groups (psychotherapy *p* = 0.021; physiotherapy *p* = 0.003; both therapies *p* < 0.001; Fisher's exact tests).

Among patients who participated in psychotherapy during the follow‐up interval (*n* = 89), diagnostic agreement was associated with greater improvement in primary FND symptoms (Wilcoxon rank‐sum *p* < 0.001, effect size *r* = 0.37; Fischer's exact *p* = 0.006 for the binary outcome). No such association was observed among those who participated in physiotherapy (*n* = 51) (Wilcoxon *p* = 0.17, *r* = 0.19; Fischer's exact *p* = 0.50) (Figure 2). Similarly, among the subset of patients who received both psychotherapy and physiotherapy (*n* = 39), diagnostic agreement was not associated with symptom improvement at 1 year (Wilcoxon *p* = 0.15; Fisher's exact *p* = 0.49). However, diagnostic agreement was associated with return to work/school across treatment groups, including those receiving psychotherapy alone (*p* = 0.021), physiotherapy alone (*p* = 0.003), as well as both psychotherapy and physiotherapy (*p* < 0.001).

## 4 Discussion

In this retrospective cohort study of 127 outpatients with FND followed for 1 year at a quaternary academic hospital, we observed improvement in symptom status and global functional outcomes within a clinic model emphasizing neuroscience‐informed education and counseling. Between baseline and follow‐up, patients pursued interventions in the community (e.g., psychotherapy and/or physiotherapy), and diagnostic agreement, symptom status, and functional outcomes were systematically assessed at 1 year. Moderate symptom improvement was reported by 40% of patients, with 44% also reporting some level of global improvement with respect to return to work/school. Improvement in symptom status and global function was strongly associated with diagnostic agreement at 1 year, whereby 66% of patients agreed and 34% expressed varying levels of disagreement. This agreement rate may appear lower than in some prior studies (Goldstein et al. 2020), which may reflect differences in measurement approach, recruitment setting, and the heterogeneity of FND subtypes represented in our cohort. Of note, illness duration and the number of symptoms at baseline were not found to be significantly associated with the main outcomes of interest, a finding consistent with a recent systematic review (Thomas et al. 2025), as well as large trials of physiotherapy for functional movement disorder (Nielsen et al. 2025) and cognitive behavioral therapy for functional seizures (Goldstein et al. 2022).

Prior research has underscored the strong relationship between diagnostic agreement and improved outcomes in FND (Greenfield et al. 2024; Nielsen et al. 2025; Goldstein et al. 2022; O'Neal et al. 2021; Duncan et al. 2014). However, to our knowledge, only two other studies have directly reported quantifiable results on this relationship, both involving functional seizures, whereby diagnostic agreement at baseline was associated with symptom improvement at 1‐year follow‐up in an outpatient neurology setting (Duncan et al. 2014) and was associated with response to cognitive behavioral therapy (Goldstein et al. 2022). The rate of reported improvement in our study appears more favorable than previous longitudinal studies, with 40% of patients reporting at least moderate symptom improvement, while 25% reported no change or worsening (Gelauff et al. 2014; O'Neal et al. 2021). This pattern may relate in part to the clinic's structured approach, which emphasized diagnostic clarity, education with an integrated formulation linking symptoms to stress and nervous system sensitization, and emphasis on recovery potential. These interventions may have helped address transdiagnostic contributors to symptom persistence such as uncertainty, symptom hypervigilance, and encountering dismissing attitudes in the healthcare system (Bailey et al. 2024; Begley et al. 2023; Edwards et al. 2023). It is possible that education, counseling, and reassurance provided in our clinic, or interventions pursued between baseline and follow‐up, contributed to the rates of symptom and functional improvement observed in this cohort. However, given the retrospective observational design, these findings should be interpreted as associations rather than evidence of a causal relationship between diagnostic agreement and outcomes.

In our cohort, 71% participated in various forms of psychotherapy and 41% in physiotherapy. Interestingly, there was no significant difference in rates of participation between those who agreed versus disagreed with the diagnosis, nor was there any significant association between participation in these treatments and symptom or work outcomes in univariate analyses (Table 2). However, patients who engaged in psychotherapy and agreed with the FND diagnosis had a strong association with symptom improvement, which was not observed for those who participated in physiotherapy. In contrast, diagnostic agreement was associated with improvement in work/school status across treatment groups. While the direction of this relationship remains unclear, it is possible that diagnostic agreement enables more effective engagement with psychotherapeutic approaches, or that perceived benefit reinforces diagnostic acceptance over time (Goldstein et al. 2022). Since the majority of the cohort participated in some form of psychotherapy, we wonder if a lack of perceived benefit reinforces doubt in the FND diagnosis for some. However, lack of pre–post evaluation in diagnostic agreement limits our ability to evaluate this complex relationship.

These findings may align with work by Saunders et al., who reported that among patients undergoing intensive inpatient neuropsychiatric rehabilitation for FND, “illness coherence” (i.e., understanding of their illness) significantly improved over the course of treatment alongside reductions in emotional distress related to the illness (Saunders et al. 2024). In the outpatient setting, neuroscience‐informed education and counseling may represent an initial step toward improving illness coherence by helping patients develop a more coherent explanatory model of their symptoms. For some, this may be sufficient to facilitate recovery, particularly if reinforced through community‐based interventions such as psychotherapy or physiotherapy. For others with more persistent symptoms and disability, more intensive multidisciplinary rehabilitation may provide repeated opportunities for reinforcing and consolidating these explanatory models. Conceptually, this process may reflect the gradual updating of maladaptive or “abnormal” priors regarding bodily symptoms, which have been proposed as a central mechanism in predictive processing models of FND (Hallett et al. 2022; Edwards et al. 2012).

Significant factors associated with diagnostic agreement in our study included a history of psychiatric comorbidities, report of any childhood trauma, and perception of having had a psychological trigger for their illness. It may be that the higher diagnostic acceptance observed in patients who had these factors is due to more of an openness and acceptance to the idea of psychological factors impacting brain networks and contributing to physical symptoms.

Although most patients in this study agreed with their FND diagnosis, a substantial portion (34%) remained in disagreement. Factors reported in the literature that may lead to difficulties with acceptance of an FND diagnosis may involve lack of supporting radiologic/laboratory abnormalities, strong views of alternative diagnoses, societal unacceptability of mind–body concepts, and individual sensitivities to psychological factors, which have also been linked to poorer diagnostic agreement and resistance (Kanaan et al. 2009; Stone et al. 2003; Whitehead et al. 2015). In our cohort, we did not find an association between abnormal brain imaging at baseline and diagnostic agreement. However, ongoing medical or neurological workup at 1 year (i.e., evaluation for an alternative cause of the patient's primary symptom complex) was associated with lower diagnostic agreement. The directionality of this relationship is unclear; continued investigations may reflect diagnostic uncertainty among patients or clinicians or may arise in response to persistent symptoms or disagreement with the diagnosis. We did not assess psychological factors that may also have contributed to diagnostic disagreement.

Given the strong cross‐sectional association between diagnostic agreement and improved outcomes in this study, including greater symptom improvement and higher odds of returning to work or school, the question arises as to whether diagnostic agreement is a modifiable factor that can be targeted through specific interventions. Targeted educational and counseling strategies likely play a crucial role in supporting diagnostic understanding and treatment engagement (Cope et al. 2021; Bailey et al. 2024; Cope et al. 2017; Dahir et al. 2024), and structured educational sessions using PowerPoint presentations and neurologist‐led discussions have been associated with increased belief in FND's treatability, hopefulness regarding recovery, and improved agreement with diagnosis, supporting their potential as scalable interventions (Fusunyan et al. 2024). Motivational interviewing may be a useful approach to support diagnostic agreement or acceptance, as it has been shown to improve treatment engagement and adherence in patients with functional seizures (Tolchin et al. 2019).

Lastly, expectations and beliefs are particularly relevant in complex neuropsychiatric conditions such as FND, where they can influence symptom burden, illness experience, and perceived impairment (McLoughlin et al. 2024; Burke et al. 2020). Expectations can directly alter sensory experiences and symptom perception, with negative expectations amplifying symptom severity, prolonging symptom duration, and increasing impairment (Van den Bergh et al. 2017; Polich et al. 2020). There may be a shared neurobiological basis between placebo effects and FND, with proposed mechanisms including placebo‐induced positive expectations deactivating hyperactive fear response centers implicated in the perpetuation of FND symptoms as well as more recent modeling of the impact of expectations influencing predictive coding models of FND (Edwards et al. 2012; Burke et al. 2020; Burke et al. 2024). While controversial, harnessing positive expectations in FND warrants further exploration.

This study has several strengths. We present data on a large cohort of FND with broad inclusion criteria, capturing the wide spectrum of FND subtypes and their associated comorbidities. Few studies have examined the influence of diagnostic agreement on clinical outcomes in FND, and our findings shed light on the important potential influence on symptom improvement and global outcomes. However, as a retrospective cohort study, there are several noteworthy limitations. Although baseline assessments were generally consistent, they were not standardized and may be subject to missing data and potentially unmeasured confounders. The lack of a baseline measure of diagnostic agreement limits our ability to assess whether it changes over time or mediates improvements observed. Relatedly, other psychological constructs related to diagnostic agreement, such as measuring level of conceptual understanding of FND symptoms (i.e., illness “coherence”), perception of control over symptoms, or perception of symptom permanence/expectations of improvement, have all been associated with outcomes in FND and were not measured in this study (Nielsen et al. 2025; Goldstein et al. 2022; Saunders et al. 2024; Sharpe et al. 2010). Additionally, return to work or school is an imperfect proxy for global functioning, as it may be influenced by economic pressures, personal choice, or external factors unrelated to clinical improvement. Future studies should incorporate validated functional outcome measures such as the WHODAS, Sheehan Disability Scale, WHOQOL‐BREF, or Work and Social Adjustment Scale (WSAS). Moreover, while all patients with FND were generally offered a follow‐up, over half (55%) of patients with an initial assessment in our cohort did not have a 12‐month follow‐up appointment. Reasons may include missed appointments, cancellations, or a patient attending follow‐up at earlier time points (e.g., 3 or 6 months) but not at 12 months. This raises possibility of selection bias, as patients who return for follow‐up may generally be the ones more likely to improve or agree with their diagnosis. Factors contributing to lack of follow‐up at 1 year were not explored in this study. Outcome assessment may have also been subject to observer bias, as the same clinician who provided the initial assessment and education intervention also conducted follow‐up evaluations. Lastly, the generalizability of findings from a quaternary neuropsychiatry clinic to other clinical settings may be limited, as patient characteristics, referral patterns, and treatment approaches in highly specialized centers may differ from those in general neurology or community settings.

## 5 Conclusions

Overall, we found that a specialized FND neuropsychiatry clinic at a quaternary center, focusing on neuroscience‐based formulation, education, and leveraging positive expectations of recovery, is both feasible and may be associated with meaningful clinical improvement. A substantial proportion of patients demonstrated improvement, and this model may enhance understanding of the diagnosis while strengthening diagnostic agreement. However, causal inferences cannot (and should not) be made. Given the strong cross‐sectional association between diagnostic agreement and both symptom improvement and return to work/school at 1‐year follow‐up, structured approaches to fostering diagnostic agreement may be a critical component of optimizing outcomes in FND. Future studies could focus on integrating tools to capture more nuanced psychological aspects of diagnostic agreement and track these measures over time to better understand the relationship with clinical outcomes. Additionally, examining factors contributing to diagnostic disagreement despite repeated education and counseling efforts remains an important avenue for future research, as improving diagnostic agreement in this population has the potential to yield important clinical benefits.

## Author Contributions

**Anthony Feinstein**: writing – review and editing. **Adriano Mollica**: conceptualization, data curation, methodology, investigation, formal analysis, visualization, writing – original draft, writing – review and editing. **Matthew J. Burke**: conceptualization, methodology, data curation, supervision, writing – review and editing, writing – original draft. **Enoch Ng**: methodology, validation, writing – review and editing. **Unaisa Bhayat**: data curation, writing – review and editing. All authors contributed to reviewing and critical editing of the final manuscript.

## Funding

M.B.’s research is supported by University of Toronto and Sunnybrook Health Sciences Centre Academic Scholars Awards.

## Ethics Statement

Data collection and dissemination were approved by Sunnybrook Research Institute's Research Ethics Board.

## Conflicts of Interest

D.L.P. has received honoraria for continuing medical education lectures in functional neurological disorder, is entitled to royalties from the sale of “Functional Movement Disorder: An Interdisciplinary Case‐Based Approach,” a textbook published by Springer, and has received honoraria from Elsevier related to “Functional Neurological Disorder, An Issue of Neurologic Clinics.” The author currently serves as paid Deputy Editor of *The Journal of Neuropsychiatry and Clinical Neurosciences*. A.M., E.N., S.N., U.B., A.F., and M.J.B. declare no conflicts of interest.

## Supporting information




**Supplementary Material**: brb371491‐sup‐0001‐SuppMat.docx

## Data Availability

Data used in this study are available upon request.
