# Supplementary material for: Diagnostic Agreement and 1‐Year Outcomes in Functional Neurological Disorder Following Neuroscience‐Informed Assessment, Education, and Counseling: A Retrospective Cohort Study
Source: Brain Behav. 2026 Jul 8;16(7):e71491. doi: 10.1002/brb3.71491 (PMC13344738; doi:10.1002/brb3.71491)
Supplement: Supplementary file 1 — Supplementary Material: brb371491‐sup‐0001‐SuppMat.docx [file BRB3-16-e71491-s001.docx]

**Supplemental Methods: Example of neuroscience-based education of functional neurological disorders for patients**

*“Your symptoms are real and not imagined or “all in your head.” They happen because of changes in how your brain is functioning, not because of damage to your brain or nerves. The brain is made up of many different networks that need to work together smoothly. When the communication between these networks gets disrupted, physical symptoms like weakness, abnormal movements, or unusual sensations can occur—even when medical tests don’t show anything structurally wrong.*

*We often explain this using the example of the amygdala, the part of the brain responsible for detecting danger. During stressful or difficult life experiences, especially when the brain is still developing, the amygdala can form extra-strong connections with other areas it’s not normally as well connected to, such as those involved in movement, sensation, and bodily awareness. These changes might not cause problems right away, but years later, another stressor, whether physical or emotional, can reactivate these old circuits, “misfiring” and producing very real symptoms.*

*Another important piece is how the brain uses attention. Our brains constantly predict what’s happening in our bodies and filter information automatically, usually without us noticing. When symptoms start, it’s natural to become very focused on them. This heightened attention can actually amplify signals coming from the body, making sensations feel stronger and more distressing, which then triggers more fear and symptoms in a vicious cycle. In FND, learning how to shift attention away from symptoms and toward meaningful activities, hobbies, or, in some cases, gradually returning to work can help calm the nervous system and reduce symptom intensity.*

*The good news is that the brain is capable of neuroplasticity, meaning it can retrain and rewire itself. Because these symptoms are due to communication problems between networks, and not permanent injury, there is real potential for improvement and recovery. Treatment starts with education and learning about the disorder you have and can also involve specialized physiotherapy and psychotherapy, all of which can help calm the brain’s threat response system, help shift the attention networks of the brain off of the symptoms, and help to restore function.”*
